# An Overview of the Etiopathogenic Mechanisms Involved in the Expression of the Oral Microbiota

**DOI:** 10.3390/clinpract15040080

**Published:** 2025-04-11

**Authors:** Ion Alexandru Popovici, Cristian Ionut Orasanu, Georgeta-Camelia Cozaru, Anita-Cristina Ionescu, Lidia Kajanto, Bogdan Cimpineanu, Anca Chisoi, Adrian Nelutu Mitroi, Ionut Poinareanu, Raluca Ioana Voda, Oana Andreea Ursica, Mihaela Butcaru Pundiche

**Affiliations:** 1 Faculty of Dentistry, Carol Davila University of Medicine and Pharmacy, 010221 Bucharest, Romania; ion.popovici@umfcd.ro; 2 Center for Research and Development of the Morphological and Genetic Studies of Malignant Pathology (CEDMOG), “Ovidius” University of Constanta, 900591 Constanta, Romania; georgiana.cozaru@365.univ-ovidius.ro (G.-C.C.); anca.chisoi@365.univ-ovidius.ro (A.C.); raluca.voda@365.univ-ovidius.ro (R.I.V.); 3 Faculty of Medicine, “Ovidius” University of Constanta, 900470 Constanta, Romania; cimpineanub@yahoo.com (B.C.); adrian.mitroi@365.univ-ovidius.com (A.N.M.); ionut_poinareanu@yahoo.com (I.P.); ursica.oana@gmail.com (O.A.U.); mihaelapundiche@yahoo.com (M.B.P.); 4 “Sf. Apostol Andrei” County Emergency Clinical Hospital, 900591 Constanta, Romania; 5 Oncological Institute “Prof. Dr. Alexandru Trestioreanu”, 022328 Bucharest, Romania; anitaionescu7@gmail.com (A.-C.I.); lidia.kajanto@gmail.com (L.K.); 6 Railway Clinical Hospital, 900123 Constanta, Romania

**Keywords:** dysbiosis, inflammation, genetics, microbiota, pathogenesis

## Abstract

**Background/Objectives**: The diversity of the oral microbiota exerts its effects in maintaining dental and overall health. The unique genetic profile of each individual influences the composition of the oral microbiota, determining susceptibility to certain diseases. The aim is to observe its role by highlighting the pathogenic mechanisms involved in oral dysbiosis and identify genetic determinism’s influence in maintaining balance. **Methods**: This study was designed as a narrative review of the oral microbiota, utilizing some of the principles and guidelines of systematic review to increase methodological rigor. We examined 121 articles such as reviews, meta-analyses, editorials, and observational studies, which met the inclusion and exclusion criteria. The inclusion criteria for studies were as follows: (1) studies that evaluated the impact of the microbiota in oral or/and systemic diseases; (2) studies that observed pathogenic mechanisms in the oral microbiota; (3) studies that evaluated the interaction of the microbiota with the immune system (4); studies that evaluated genetic implications in the microbiota. **Results**: Host genes regulate inflammatory and immunological reactions that play a role in microbiological balance. This explains the increased resistance of some to diseases, including gingivitis or periodontitis. Also, the implications of oral dysbiosis are reflected not only locally, but also generally, being associated with various systemic conditions. **Conclusions**: Understanding the pathogenic mechanisms and genetic determinants involved in oral dysbiosis may help create individualized therapies for preventing and managing oral and systemic disorders. A healthy lifestyle and adequate oral hygiene can facilitate a diverse and balanced microbiome, crucial for overall health.

## 1. Introduction

An intriguing and emerging area of research is examining the microbiota and its interactions with the host genome. Understanding the interactions between the human genome and the microbiota, the vast community of microbes that live in and on the human body, helps us identify the processes by which various disease disorders arise, evolve, and occasionally can be avoided [1,2,3]. The human microbiota, found mainly in the digestive tract, but also on body surfaces and in the respiratory tract, represents a diverse ecosystem of bacteria, viruses, fungi, and other microorganisms [1]. Our health depends on this community of microorganisms. They help digestion, metabolism, immune balance, and even cognitive or behavioral patterns. Thus, the human body provides a suitable environment for them, in an excellent symbiotic connection, providing several health benefits [1,2,3,4].

The oral microbiota is a complex community comprising approximately 1900 phylotypes, most of which are uncultivable. The evolution over time and the interaction between them are essential components of the balance between health and disease [5,6]. Until recently, the oral microbiota of the fetus in utero was thought to be sterile. However, strains of *Fusobacterium nucleatum* could be cultivated. This strain has been associated with periodontal disease, which in some cases may be a risk factor for premature birth and low-birth-weight babies [5]. Subsequently, several factors shape the oral microbiome in children. These include genetics, mode of delivery, and type of nutrition, breastfeeding, or formula [6].

From birth, the oral cavity comes into contact with numerous pathogens. In the case of natural birth, the bacterial agents are represented by *Lactobacillus*, *Prevotella*, and *Sneathia* spp. In the case of dystocia births, *Staphylococcus*, *Corynebacterium*, and *Propionibacterium* spp. predominate. As babies grow, the microbial community evolves, and infants (around 5 months of age) develop their microbiota, different from that taken over from the mother. This microbiota consists mainly of bacteria, including the six phyla: *Firmicutes*, *Proteobacteria*, *Actinobacteria*, *Bacteroidetes*, *Fusobacteria*, and *Spirochetes*. Tooth eruption is an important event in terms of the appearance of cariogenic species (*S. mutans*) [5,7].

The first years of life are the most important for the diversification of the oral community. During this time, the oral biofilm begins to self-assemble, reaching over 32 taxa at the species level at approximately two years of age. Breastfed infants have a higher abundance of oral bacteria, with breast milk playing a prebiotic role. The introduction of solid foods increases the heterogeneity of the oral microbiome regardless of the type of feeding (breastfeeding or formula) [6]. As the dentition becomes permanent, the population of bacteria belonging to the *Veillonellaceae* family and the *Prevotella* genus increases, while bacteria from the *Carnobacteriaceae* family decrease. Hormonal changes at puberty are also reflected in the oral microbiota. Thus, the group of anaerobic Gram-negative bacteria and spirochetes increases, explaining the severity of gingivitis during this period. Regarding other types of agents, studies have shown that among fungi, *Candida* is the most common, decreasing with age, and among viruses, the most common are bacteriophages [5,8].

The terms eubiosis and dysbiosis describe two opposing states of microbiota balance that reflect the impact of the microbiome on host health [1]. Eubiosis refers to a state of equilibrium in which the microbiota is diverse and well adapted to perform its functions of protection, digestion, and immune support. Commensal and mutualistic microbes predominate in eubiosis, thus preventing colonization by pathogens. When this balance is disrupted, dysbiosis results, a phenomenon in which pathogenic or opportunistic species thrive [1,2]. Some current studies indicate that some genetic polymorphisms may provide a more suitable habitat for certain types of microbes, thereby affecting the composition of the microbiota in a way that could have long-term consequences on health [1,2]. Some genes play a role in regulating the pH or chemicals found in a particular tissue or organ, affecting environmental adaptation for certain microbial species [2]. Likewise, gene expression can be influenced by procedures such as chemical exchange and gene transfer, thus affecting our health. Subsequently, the host genome can simultaneously affect the structure and functionality of the microbiota. These alterations interact in a complex bidirectional manner [1,2,3].

Given the paucity of literature on the implications of genetic determinism in the oral microbiome, the purpose of this study is firstly to observe the role of the oral microbiota by highlighting the main pathogenic mechanisms involved in oral dysbiosis, and secondly to identify the influence of genetic determinism in maintaining the balance of the microbiota. Through this study, we want to highlight the interaction between genetics and oral microbiota to establish a foundation for future studies on personalized treatment.

## 2. Materials and Methods

**Figure 1:**
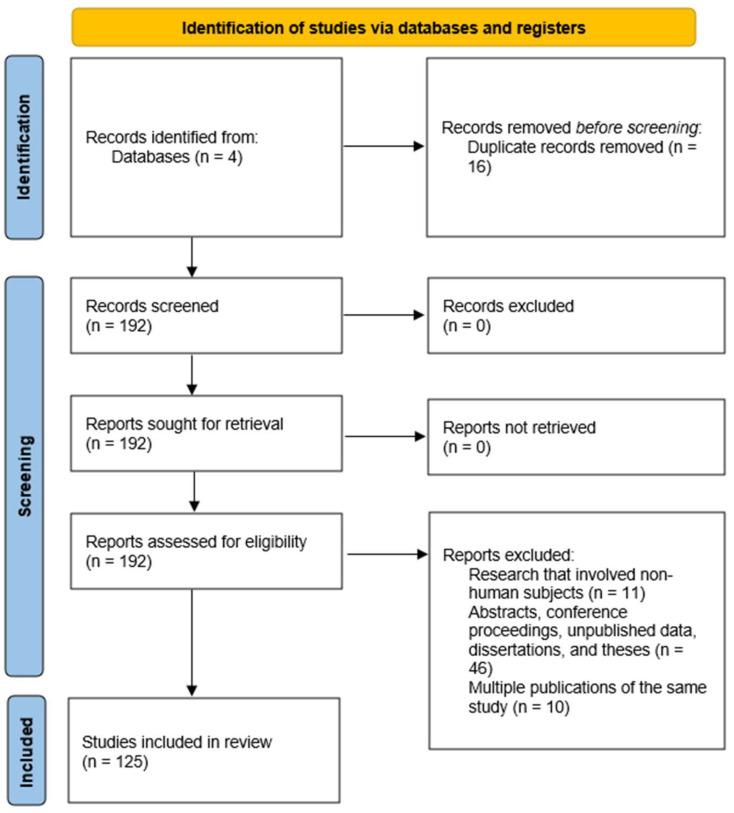
PRISMA flow chart outlining the process of identifying, screening, and including studies for this review on oral microbiota. From the 4 databases studied, 192 articles entered the screening process, of which 125 met the study eligibility criteria.

This study was designed as a comprehensive narrative review of the oral microbiota. However, some of the principles and guidelines of systematic review were followed and applied to increase methodological rigor. The PRISMA criteria were adapted to provide a transparent and structured approach for identifying, screening, and including relevant studies. A literature review was performed to identify the pathogenic mechanisms involved in oral dysbiosis and the genetic implications in the oral microbiota. Given the paucity of studies, especially analytical ones (observational and experimental), with clear evidence of the impact that genetic determinism has on the oral microbiota, we opted for the narrative nature of the study. Therefore, the PICO criteria cannot be applied fairly. Instead, the following question may arise: “Can genetic determinism affect the oral microbiome through pathogenic mechanisms of dysbiosis?”. Four main databases—MEDLINE (PubMed), Web of Science Core Collection, Google Scholar, and Science Direct Collection Elsevier—were searched comprehensively for literature (Figure 1). The search was performed from 12 to 31 December 2024 and included publications from 1 January 2010 to 1 December 2024. The search was conducted using targeted keyword matching to ensure a comprehensive and focused approach to gathering relevant information. Medical Subject Headings (MeSH) terms were used. Main search terminology used: “microbiota”, “oral”, “pathogenesis”, “dysbiosis”, and “genetics”.

### 2.1. Eligibility Criteria

The inclusion criteria for studies were as follows: (1) research assessing the influence of the microbiome in either oral or systemic disorders; (2) research noting pathogenic processes within the oral flora; (3) research assessing the relationship between the immune system and the bacteria; (4) studies assessing genetic implications in the oral microbiota.

The exclusion criteria encompassed the following: (1) research that involved non-human subjects; (2) abstracts, conference proceedings, case reports, unpublished data, dissertations, and theses that were not peer-reviewed full-text articles; (3) multiple publications of the same study (the most comprehensive version was included).

### 2.2. Selection Process

The first step in the screening process was the examination of the titles and abstracts of the papers. At this point, studies failing the inclusion criteria were deleted. Under I.A.P.’s direction, L.K., B.C., I.P., and O.A.U. first screened titles and abstracts. Using the established inclusion criteria, they evaluated the relevance of the studies through the examination of their abstracts.

We received and closely examined the entire text of every item that passed the first screening. This phase guaranteed that every included study satisfied the required criteria and supplied enough information for examination. Examining each article for alignment with the inclusion criteria, including study design (reviews, meta-analyses, editorials, observational studies), participant characteristics (if present), interventions, outcomes, and the quality of the research. C.I.O., G.C.C., A.C.I., and A.C. were responsible for the thorough full-text review. The final list of included studies was validated by I.A.P., R.I.V., and O.A.U., therefore guaranteeing a strict selection consistent with the goals of this narrative review. Discrepancies or uncertainties identified during the full-text review were addressed through group discussion, with P.B.M. guaranteeing methodological rigor. B.C., A.N.M., and P.B.M. reviewed clinical relevance and applicability. I.A.P., L.K., I.P., and R.I.V. formally reviewed quality assessments to detect potential effects on research results and robustness using the Critical Appraisal Skills Program (CASP) checklists. The manuscript’s grammar and syntax were reviewed using QuillBot (Version 18.26.0) to enhance clarity and readability.

## 3. Implications of the Oral Microbiota in Systemic and Dental Diseases

The oral microbiota is a multifaceted collection of microorganisms. It encompasses the balance-oriented bacteria, fungi, viruses, and protozoa that help maintain oral health [1,2]. Oral dysbiosis is an imbalance that may favor the growth of pathogenic bacteria at the expense of good ones, thereby causing oral disorders (Table 1) and a detrimental effect on overall health (Table 2). Dental caries, gingivitis, periodontitis, and opportunistic infections are among the many pathogenic diseases resulting from this imbalance. Factors such as poor oral hygiene, a diet high in carbohydrates, antibiotic use, stress, or systemic diseases help to detect this change in the microbial environment.

**Table 1:** Metagenomic studies of associations between the oral microbiome and oral diseases.

Oral Disease	Study Focus	Key Findings	Implications
**Periodontitis**	Dysbiosis of subgingival plaque [4]	Increasing prevalence of *Treponema denticola*, *Tannerella forsythia*, and *Porphyromonas gingivalis*. Mechanisms for biofilm formation and sulfur metabolism.	Discovered possible diagnoses and therapy paths as well as microbiological indicators.
**Dental caries**	Microbial diversity in caries severity [9]	Enrichment of *Streptococcus mutans*, *Lactobacillus* spp., and *Bifidobacterium dentium*. Less diversity and more acidogenic metabolism.	Ideas for preventative plans emphasizing microbial diversity restoration and acidogenic activity.
**Oral cancer (OSCC)**	Role of *Fusobacterium nucleatum* in cancer development [10]	*F. nucleatum* enriched in tumors. Activation of NF-κB pathways, immune evasion, and resistance to apoptosis.	Highlights *F. nucleatum* as a therapeutic target in oral squamous cell carcinoma.
**Halitosis**	Microbial dysbiosis in tongue biofilm [11]	Increased *Solobacterium moorei*, *Prevotella* spp., and *Fusobacterium nucleatum*. Elevated sulfur-reduction pathways.	Discovers bacterial causes of halitosis for focused treatments.
**Oral lichen planus (OLP)**	Altered microbiome in buccal mucosa [12]	Decreased *Streptococcus salivarius*, increased *Prevotella* spp., and *Porphyromonas* spp. Upregulation of LPS biosynthesis and immune modulation genes.	Suggests a microbial involvement in the oral lichen planus inflammatory condition.
**Diabetes and periodontitis**	Salivary microbiome in comorbidities [13]	Enrichment of *P. gingivalis* and *T. denticola*. Increased oxidative stress and pro-inflammatory cytokine activation pathways.	Emphasizes oral-systemic health relationships and inflammatory routes.
**Oral candidiasis**	Fungal-bacterial interactions [14]	Co-occurrence of *Candida albicans* and *Streptococcus mutans*. Enhanced biofilm formation and quorum sensing.	Clarifies the function of microbial collaboration in biofilm resistance and degree of infection.
**Sjögren’s Syndrome**	Altered salivary microbiome [15]	Reduced microbial diversity. Increased *Lactobacillus* species, decreased *Streptococcus*. Altered oxidative stress and immune signaling pathways.	Makes recommendations on microbial dysbiosis in inflammation and autoimmune.

In addition to affecting the state of the oral cavity, the oral microbiota has broad consequences for overall systemic health [3]. For example, metagenomics has shown that some bacterial species, such as *Fusobacterium nucleatum* or *Porphyromonas gingivalis*, are involved in the development of disorders such as colorectal cancer, Alzheimer’s disease, or diabetes mellitus (Table 2) [16,17,18,19,20,21]. Research on cardiovascular disease has also shown that bacterial deoxyribonucleic acid (DNA) from the oral microbiome (e.g., *Porphyromonas gingivalis*) is present in atherosclerotic plaques (Table 2). This implicates a possible process by which oral infections cause systemic inflammation and vascular wall dysfunction.

**Table 2:** Metagenomic studies of associations between the oral microbiome and systemic diseases.

Systemic Disease	Study Focus	Key Findings	Implications
**Cardiovascular disease**	The link between *Porphyromonas gingivalis* and atherosclerosis [16]	Detection of *P. gingivalis* DNA in atherosclerotic plaques. Association with inflammation pathways and vascular damage.	Implies that oral bacteria cause systematic inflammation and vascular malfunction.
**Diabetes mellitus**	Oral microbiome shifts in type 2 diabetes [17]	Reduced microbial diversity. Increased abundance of *Prevotella intermedia* and *Porphyromonas gingivalis*.	Emphasizes how oral dysbiosis affects glucose metabolism and general inflammation.
**Adverse pregnancy outcomes**	Association of oral bacteria with preterm birth [18]	*Fusobacterium nucleatum* detected in the placenta of preterm birth cases. Bacteria are linked to inflammation and premature rupture of membranes.	Proposes translocation of oral bacteria as a risk factor for negative pregnancy results.
**Alzheimer’s disease**	Role of *P. gingivalis* in neurodegeneration [19]	*P. gingivalis* and its gingipain enzymes detected in brain tissue of Alzheimer’s patients. Associated with amyloid plaque formation and neuroinflammation.	Proposes oral infections as possible causes of neurological diseases and treatment targets.
**Rheumatoid arthritis**	Oral dysbiosis and autoimmune activation [20]	Elevated *Aggregatibacter actinomycetemcomitans* in patients. Association with hypercitrullination and autoantibody production.	Supports the role of oral bacteria in triggering autoimmune responses in rheumatoid arthritis.
**Colorectal cancer**	Impact of *Fusobacterium nucleatum* on colorectal tumor progression [21]	*F. nucleatum* promotes tumor growth via E-cadherin/β-catenin signaling. Detected in higher abundance in colorectal tumor tissues.	Highlights *F. nucleatum* as a potential biomarker and therapeutic target for colorectal cancer.
**Chronic kidney disease**	Oral microbiome changes in chronic kidney disease [22]	Increased abundance of *Tannerella forsythia* and *Fusobacterium nucleatum*. Association with systemic inflammation and uremic toxins.	Suggests that oral dysbiosis may exacerbate inflammation in chronic kidney disease.
**Respiratory infections**	Oral microbiome in aspiration pneumonia [23]	Increased prevalence of *Streptococcus pneumoniae* and *Pseudomonas aeruginosa* in oral samples of patients with pneumonia.	Highlights the oral cavity as a reservoir for respiratory pathogens in vulnerable individuals.

## 4. Mechanisms of Pathogenesis

The mechanisms of pathogenesis in oral dysbiosis are complex, with multiple components, including interactions between oral bacteria, the host immune response, and environmental factors [24,25,26,27,28]. The microbial balance, as well as the host status, are affected when conditions in the oral cavity allow the proliferation of harmful bacteria—dysbiosis [4]. The main pathogenic processes of oral dysbiosis are described below.

### 4.1. Alteration of the Microbial Equilibrium

Disturbance of balance is one of the key processes that causes oral dysbiosis. A wide variety of microbial species coexist in symbiosis with the host and form the healthy oral flora. Usually, through processes of competition for resources with pathogens and by generating antibiotic substances, which stop the spread of dangerous bacteria, beneficial bacteria help maintain a balanced oral environment [23,24,25,26,27,28,29]. Some pathogenic species (*Porphyromonas gingivalis*, *Fusobacterium nucleatum*, or *Streptococcus mutans*) replace beneficial bacteria when the oral microbiota is disturbed by an unbalanced diet (excessive sugar consumption), poor hygiene, or excessive use of antibiotics. The consequences are the production of inflammation and oral diseases such as gingivitis, periodontitis, or dental caries [30,31].

### 4.2. Biofilm Production

Antimicrobial therapies lose their effectiveness when bacterial biofilms create a protective extracellular matrix, which isolates microorganisms from environmental stress and the host immune system. Pathogens such as *Streptococcus mutans* and *Porphyromonas gingivalis* utilize biofilms, creating a persistent inflammation that guarantees the survival of the bacterial community [32]. The processes of biofilm development consist of bacterial adherence, extracellular matrix synthesis, and bacterial community maturation. These are governed by host genetic elements involving oral surfaces. Chronic inflammation is triggered by the production of toxins and metabolites by mature biofilms. This process fuels periodontal disease and caries [32,33]. Adherent biofilms are formed by bacterial colonies (particularly *Porphyromonas gingivalis*, *Treponema denticola*, *Fusobacterium nucleatum,* and *Streptococcus mutans*) and are embedded in a matrix of extracellular compounds (polysaccharides, proteins, nucleic acids) that protect them from immune and antimicrobial responses. They cause inflammation of the gums and are difficult to eradicate [32,33,34].

Biofilm formation begins both with the attachment of bacteria to tooth surfaces using specialized adhesion structures (pilus, fimbriae) and attraction to salivary components (glycoproteins). Initially, the bacteria generate extracellular compounds that help develop the protective matrix. Subsequently, the biofilm develops and stratifies over time to create complex colonies (including multiple species). These dysbiosis colonies consist mostly of pathogenic bacteria, which produce enzymes and toxins that promote inflammation and damage oral tissues. An effective barrier against antimicrobial agents and the immune response is the extracellular matrix [32,33,34]. Immune system cells, neutrophils, and macrophages have difficulty penetrating the biofilm to phagocytize bacteria. Also, the action of antimicrobials (antibiotics and antiseptics) in oral hygiene products may be reduced because the matrix reduces their permeability [34]. Another consequence is the formation of dental plaque. This originates from biofilm, which is a dense aggregation of harmful bacteria and organic elements deposited on the surface of teeth and gums [32].

Using a technique called “quorum sensing”, the bacteria in the biofilm communicate and coordinate their activity. This helps them in the processes of tissue degradation, worsening inflammation and gum lesions. Another effect is the synchronization of toxin and enzyme synthesis. Thus, bacteria exchange genetic material that may contain resistance genes, providing group protection against treatments and helping to consolidate and spread antimicrobial resistance mechanisms [35,36,37,38].

### 4.3. Toxin Release and Proteolytic Enzyme Production

Toxins produced by pathogenic bacteria in the oral cavity directly influence the inflammatory process and stimulate tissue destruction. *Porphyromonas gingivalis* and other pathogenic bacteria in the oral biofilm, through cytotoxic proteins, destroy gingival tissues and can generate periodontal ulcers [4,30]. Gingipains (produced by *P. gingivalis*) or dentilisin (produced by *Treponema denticola*) degrade collagen and structural components of the gum and alveolar bone. The toxins also affect the host’s immune system, producing overactive responses that lead to chronic inflammation [4]. They stimulate pro-inflammatory cytokines (IL-1β) accelerating periodontal tissue damage and intensifying the inflammatory process. Toxins can also disrupt dental mineral homeostasis by generating organic acids that demineralize enamel.

*T. forsythia*, *T. denticola*, and *P. gingivalis* are becoming the most widespread pathogenic bacteria in the oral microbiota and are the main ones involved in periodontal tissue damage [30,34,35]. The toxins and proteolytic enzymes they produce aggravate chronic inflammation and progressive destruction of the gums, periodontal ligaments, and alveolar bone. Thus, they are directly involved in severe gingivitis and periodontitis [30]. These gingipains are essential for the pathogenicity of the bacteria and represent a source of toxins that degrade the structures surrounding the periodontal tissues. Moreover, *Treponema denticola* produces proteases (such as dentilisin and Msp protein) that have synergistic effects in attacking tissues and supporting the progression of inflammation [39,40].

Proteolytic enzymes and toxins influence periodontal structures as follows: (1) collagenases weaken gingival collagen and periodontal ligaments, causing loss of tooth-gingival attachment; (2) bacterial toxins activate osteoclasts and cause alveolar bone loss. They are also involved in the development of periodontal pockets suitable for the spread of anaerobic bacteria [39,40,41,42].

### 4.4. Modification of the Oral Mucosal Barrier

The integrity of the oral mucosa plays a critical role in protecting the body against microbes. A significant impact on this protection is exerted by host genes that modulate immune responses and epithelial integrity. Genetic variations that influence the activity of epithelial cell-binding proteins, such as cadherins and cytokines, can make the oral mucosa more permeable to pathogenic bacteria [43]. Thus, oral dysbiosis can compromise the oral mucosal barrier, allowing bacteria to pass into deeper tissues and into the circulation.

### 4.5. Chronic Inflammation and Systemic Impact

Another crucial process associated with oral dysbiosis is the inflammatory response. Pathogenic bacteria in the oral cavity can trigger a local immune response that, if chronic, causes tissue degradation, including the gingiva and alveolar bone [4,32]. In addition to the local impact, chronic inflammation of the oral cavity also has systemic consequences. In the context of oral dysbiosis, inflammatory substances (cytokines and interleukins) can reach the blood and stimulate the development of cardiovascular diseases, diabetes mellitus, respiratory diseases, or even neurodegenerative disorders [4,28,44]. Lipopolysaccharides released by Gram-negative bacteria (*P. gingivalis*) enter the bloodstream and can cause the development of atherosclerotic plaques [44]. Constant systemic inflammation decreases insulin sensitivity, thus worsening the symptoms of diabetes. At the same time, hyperglycemia promotes the proliferation of pathogenic bacterial species, exacerbating periodontitis [13].

Proteolytic enzymes promote inflammation by encouraging the immune system to produce proinflammatory cytokines such as tumor necrosis factor-alpha (TNF-α), interleukin-1 (IL-1), and interleukin-6 (IL-6). One of the first mediators generated in response to bacterial infections is interleukin-1 beta (IL-1β). It is necessary to activate the processes of collagen decomposition and the activity of osteoclasts, causing the loss of alveolar bone. TNF-α has a high pro-inflammatory effect, triggering tissue degradation processes and attracting other inflammatory cells to the affected tissues. IL-6 promotes the immune response and causes damage to periodontal tissues by favoring osteoclast activity, promoting bone loss.

Apart from cytokines, lipopolysaccharides (LPS) cause the activation of neutrophils and macrophages. These cells generate reactive oxygen species and proteolytic enzymes. In excess, they cause the degradation of collagen and other gingival structures [45]. Tissue destruction and chronic inflammation provide a breeding ground for pathogenic bacteria that infiltrate the affected areas and proliferate in local biofilms. This anaerobic environment produced in periodontal pockets favors the proliferation of certain pathogenic bacteria, sustaining a vicious cycle in which inflammation supports the growth of pathogenic bacteria, which in turn exacerbates inflammation and tissue destruction [46,47,48].

### 4.6. Dysfunctions of the Host Immunological Response

Malfunctioning of the host immune response is another crucial mechanism that underpins oral dysbiosis. The reduced ability to combat harmful microorganisms may result from genes that control the immune system, particularly those that regulate the activity of T cells, B cells, and other components of immunity [49]. Some variations in genes that encode cytokines can cause either an excessive or inappropriate inflammatory response. This allows harmful bacteria to thrive more easily in the oral cavity and leads to chronic disorders such as gingivitis or periodontitis [50,51].

### 4.7. Interaction Between Oral Microbiota and Environmental Factors

Oral dysbiosis can be influenced by diet (consumption of refined carbohydrates and sugars), smoking, antibiotics, and stress. The response is generated either by increasing the number of pathogenic bacteria or by modifying the host’s immunological responses. Carbohydrates provide a substrate for cariogenic bacteria, which generate acid and lead to the formation of dental caries. Smoking decreases immune system function, allowing bacteria to establish themselves more easily in the oral cavity [52,53]. Smoking also causes immunological suppression, oxygen deprivation, and biofilm development. The result is a decrease in beneficial oral bacteria and the appearance of oral disorders [54,55]. Studies have shown that, regardless of sampling methods or locations, certain microbial species are more prevalent in smokers compared to nonsmokers. Specifically, cultures from smokers have demonstrated lower levels of *Neisseria* and *Branhamella* species [54,56]. Mason et al. compared subgingival plaque samples from 200 smokers and nonsmokers and observed notable variations in microbial profiles [56]. Smokers were shown to have a more diverse microbiome, rich in pathogens and lacking in commensals. This microbial composition more closely resembles that of a disease-associated community, even in clinically healthy individuals. Aspect indicates that smoking creates a precarious environment that leads to disturbances in the oral microbiome [54,55,56,57]. Dairy products, fruits, and vegetables can create a more alkaline environment in the mouth that could inhibit the growth of pathogenic bacteria and reduce the risk of gingivitis [52,53]. Pollutants can alter the gene expression of oral microorganisms, inducing oral tissue damage and chronic inflammation [4,15,24]. Additionally, regular and unregulated use of antibiotics may destroy commensal bacteria, supporting the spread of pathogenic microbes. Other medications, such as antidepressants and antihistamines, may reduce salivary flow, promoting the growth of harmful germs [58,59,60,61]. Probiotics and prebiotics administered to rebalance the gut microbiota have been studied for their beneficial effects on oral health. Some strains of Lactobacillus and Bifidobacterium also have beneficial effects on the bacterial balance in the oral cavity, helping to prevent cavities and periodontal disease [62,63]. Recent research has examined the gut-brain axis in people with alcohol use disorder, with or without alcoholic liver disease. The compromised gut barrier allows bacterial products to enter the circulation and triggers central inflammation [55,57].

## 5. The Contribution of Endogenous Factors in the Development and Occurrence of Oral Dysbiosis

While hundreds of projects worldwide are in development and research on the human microbiome has seen notable growth, the oral microbiome has not been as well investigated as the gut microbiome [1,64]. Genetic determinism plays a role in the development and maintenance of the oral microbiota throughout life. This modulates its stability and diversity. Host genes intervene in different phases of the interaction between oral bacteria and the immune system, maintaining microbial balance [65].

Each person’s genetic makeup affects the composition and function of the oral microbiome, influencing the types and abundance of microbial species that colonize the oral cavity. Thus, some individuals may be more prone to developing periodontitis, while others may have a balanced response to oral dysbiosis [66]. Therefore, understanding how genetic elements influence the oral microbiome opens up an interesting avenue of study that could allow for the development of individualized therapies for oral diseases. Furthermore, it could shed light on how microbiota variations could cause or aggravate systemic diseases (diabetes, cardiovascular diseases, or neurodegenerative diseases).

In the last ten years, thanks largely to the development of innovative genomic technologies, Next-Generation Sequencing (NGS), and advanced bioinformatics tools, major advances have been made in our knowledge of the critical function of the oral microbiome. NGS—which encompasses a variety of methods including 16S rRNA sequencing—metagenomics, shotgun metagenomics, and quantitative real-time PCR (RT-PCR) have revolutionized microbiome research by replacing traditional culture-based techniques. These cutting-edge methods allow for a much more precise and detailed investigation of the complex structure and function of the microbiome, providing insights previously impossible [66,67,68].

Among the many methods, stimulated saliva collection has become a useful tool for oral microbiota research. This approach provides a regulated environment ensuring that the data obtained are thorough and fairly representative of the microbiome present in the oral cavity. Stimulated saliva represents a mixture of microbial species from different parts of the oral environment. This allows researchers to gain a more precise understanding of the diversity of the microbiome, its functional roles, and how it interacts with the host [68,69]. This method has opened up new opportunities for research into microbial ecology and provided insightful analysis of how the oral microbiome influences many different health outcomes. Furthermore, these developments pave the way for the creation of more focused treatments, personalized health plans, and preventive actions aimed at restoring or preserving the fragile balance of the oral microbiota, thereby improving oral and overall health [66,67,68,69].

Such studies could completely transform the way we preserve or repair oral and systemic health. Targeted treatments designed to restore microbial balance or stop disease before it starts could be developed by understanding the genetic markers that predispose people to certain oral health problems. Moreover, as our knowledge increases, we could find creative approaches to alter the microbiome, using dietary changes, probiotics, or other therapies to improve health outcomes. Integrating microbiome science with genetics may result in more personalized health care approaches that result from the delicate interaction between genes and microbial ecosystems [69,70,71,72].

### 5.1. Oral Microbiota and Immune System Interactions

Variability in cytokine receptor genes may influence the body’s response to oral infection. Deficiencies in the expression of TLRs (Toll-like receptors) may result in an inadequate immunological response, encouraging the spread of pathogenic bacteria [73]. Polymorphisms in genes encoding proinflammatory cytokines could stimulate the local inflammatory response, increasing the risk of periodontal disease. Essential components in the inflammatory cascade, these genes are fundamental for the generation of proinflammatory mediators, as well as for the activation of immune cells, including macrophages and neutrophils [71]. In those with particular polymorphisms in IL-1β or TNF-α, the inflammatory response may be disproportionately strong, leading to a persistent inflammatory state. Increased bone resorption, tissue damage, and slow tooth loss may result from this chronic inflammation [71,72]. For example, genetic diversity of IL-1β receptors may influence the degree of immune response and severity of periodontal disease. Thus, the immune system may overreact to oral bacteria, resulting in tissue damage and disease progression [72,73].

A crucial element in the identification of pathogen-associated molecular patterns (PAMPs) is the expression of Toll-like receptor (TLR). The initiation of the innate immune response and the subsequent direction of adaptive immunity depend on it. Their deficiencies can lead to an inadequate immune response, thus allowing dangerous microorganisms to multiply unchecked. The inability to identify and respond appropriately helps pathogenic bacteria to establish and flourish in the oral cavity, aggravating the oral inflammatory state and causing periodontal disease [73,74,75].

These genetic elements highlight the intricacy of the immune response of the host in periodontal disease and show how differences in immune system genes could affect a person’s sensitivity to oral infections. Understanding these genetic predispositions might help to create more customized prevention and treatment plans for periodontal disease, emphasizing either changing the immune response or fixing particular genetic defects to prevent too strong inflammation and tissue damage [72,73,74,75].

The body’s ability to manage reactive oxygen species (ROS) and protect tissues from oxidative damage critically depends on genes that control xenobiotic metabolism and oxidative stress. These genes are part of the body’s defense mechanisms against oxidative stress, which are key elements in the development and maintenance of chronic inflammation. Thus, the control of oxidative stress critically depends on genes such as glutathione S-transferase pi 1 (GSTP1) and superoxide dismutase 2 (SOD2). GSTP1 is involved in the detoxification of oxidative products, allowing the conjugation of glutathione with reactive chemicals, protecting cells from oxidative damage. SOD2 is essential in the transformation of toxic superoxide radicals into less harmful molecules [76].

In periodontitis, there is increased oxidative stress. Therefore, the body’s ability to control this stress directly influences the degree of destruction of periodontal tissue. Individuals with genetic variants that compromise the action of antioxidant enzymes (SOD2 or GSTP1) show increased oxidative damage in periodontal tissues, increasing their susceptibility to inflammation and tissue degradation [74,76].

Oxidative stress and periodontal disease highlight a complex relationship between hereditary factors and environmental influences (nutrition, smoking and oral hygiene). Understanding how oxidative stress can be controlled and how it varies provides important information for individualized oral health care strategies. Targeted treatments through antioxidant therapies could be developed to reduce its effects. It is also necessary to identify individuals with increased hereditary risk in order to protect periodontal tissues [74,75,76,77].

### 5.2. Genetic Regulation of Collagen Synthesis and Degradation in Oral Health

Found in the composition of dental tissues (dentin, periodontal ligament and alveolar bone), collagen is a major structural protein that helps teeth to be stable and functional. Given its relevance, the genetic control of collagen turnover (synthesis, function and degradation processes) has attracted major attention in recent years, being implicated in both general health and pathological processes [78]. Collagen synthesis is a highly regulated process involving a sequence of enzymatic steps and genetic control. COL1A1 and COL1A2 are key genes, encoding the α1 and α2 chains of type I collagen, respectively [79]. Type I collagen is the most abundant type of collagen in bone and dental tissues, providing a framework for strength and rigidity. Variations or mutations in these genes result in altered collagen production and compromise the structural integrity of dental tissues. It can also lead to disorders such as osteogenesis imperfecta (brittle bone disease), which can also affect teeth [80]. Not only is collagen turnover limited to production, but the maintenance of tissue homeostasis also depends on the breakdown of collagen fibers [79,80]. A family of enzymes crucial in collagen degradation are the matrix metalloproteinases (MMPs). MMP-1 (collagenase) degrades the collagen triple helix, allowing tissue remodeling and healing. The maintenance of connective tissue health, including the periodontium, depends on a balance between collagen synthesis and degradation [81].

One consequence of variations in genes encoding structural proteins and enzymes is an alteration in the balance of collagen turnover. Thus, MMP-8 mutations have been associated with a higher risk of periodontal disease [82,83]. Analogous changes in the COL1A1 gene may compromise the structural stability of dental tissues, increasing susceptibility to both dental caries and periodontal disorders [84].

The alveolar bone is constantly remodeling in response to mechanical stress and inflammation. A major component of the bone matrix is collagen. Its role is crucial for its development and resorption [85]. Bone resorption is regulated by polymorphisms in genes such as osteoprotegerin (OPG) and receptor activator of nuclear factor kappa-B ligand (RANKL). These can affect collagen turnover in bone tissue and contribute to disorders including osteoporosis and periodontitis [86]. In periodontal disease, an imbalance between collagen degradation and synthesis in the periodontal ligament can cause alveolar bone resorption and loss of tooth attachment. Studies have indicated that those with certain polymorphisms in COL1A1 or MMP may be more likely to experience more severe bone loss as periodontal disease progresses.

In addition to its function in bone metabolism, collagen is a necessary component of dentin, the hard tissue beneath the enamel. Collagen fibers form a framework for the deposition of minerals, which define the integrity of dentin. Changes in collagen genes may affect dentin synthesis, leading to disorders including dentinogenesis imperfecta [87]. Weak, discolored, and brittle teeth are produced by this inherited condition. A similar breakdown of collagen fibers in the periodontal ligament, leading to loss of support and attachment of teeth, defines periodontal disorders.

### 5.3. Genes of Lipid Metabolism in the Context of Oral Dysbiosis

Maintaining metabolic balance and the regular functioning of the body depends on lipid metabolism. Several aspects of health, including inflammatory reactions and dysbiosis in the oral flora, have been linked to genes involved in this process [88]. In this regard, the peroxisome proliferator-activated receptor gamma (PPARγ) is among the most vital genes, as it is essential for the control of lipid metabolism and the inflammatory response [89]. This gene belongs to the family of nuclear receptors that control the expression of genes involved in the metabolism of fatty acids and triglycerides, thus affecting the metabolic state of the body and, consequently, oral health. PPARγ controls insulin sensitivity, as well as other steps necessary for lipid metabolism, including the accumulation and utilization of triglycerides. Including in the oral cavity, its activity is closely correlated with local and systemic inflammation. Variations in PPARγ activity could affect metabolic balance, favoring an altered metabolic profile that influences the oral microbiota and favors the proliferation of pathogenic bacteria [89,90].

Chronic inflammation and loss of supporting tooth tissues have been linked to pathogenic bacteria in the oral cavity, including *Tannerella forsythia*, *Fusobacterium nucleatum*, and *Porphyromonas gingivalis*. An altered metabolic profile whereby lipid metabolism is unregulated can cause changes in the local immune system, therefore encouraging the spread of certain harmful bacteria. Furthermore, aggravating immunological reactions to these infections is persistent inflammation triggered by PPARγ and lipid metabolism, destroying periodontal tissues and advancing periodontitis [89,91].

### 5.4. Epigenetic Changes Induced by Pathogenic Microbiota

Oral dysbiosis can induce significant molecular changes largely through epigenetic processes [92]. These changes can affect host gene expression and lead to pathogenic disorders, including malignant transformation. Within the oral microbiota, the epigenetic mechanisms involved—DNA methylation and histone modifications—can have a major impact on oral and overall health [93]. DNA methylation is one of the best-studied epigenetic processes through which oral dysbiosis may influence host cells. This is a process by which methyl groups are added to cytosine, altering gene expression without altering the DNA sequence. Tumor suppressor genes or those involved in cellular self-repair can be kept under control by this method [94]. Studies of some pathogenic bacteria in the oral cavity, including *Porphyromonas gingivalis*, have revealed associations with aberrant DNA methylation [95]. These bacteria can alter methylation patterns, which suppresses tumor suppressor genes. Through this aberrant methylation, *P. gingivalis* indirectly helps to decrease the ability of cells to defend themselves against malignant changes. Furthermore, excessive methylation of certain suppressor genes could lead to dysregulation of cell proliferation and programmed cell death processes, thus encouraging the growth of malignant tumors. Vyhnalova T. et al., revealed that pathogenic bacteria in the oral cavity could interact with host mechanisms and encourage malignant transformation in oral tissues through epigenetic changes [96,97]. These results highlight the need for microbiotic balance and how disruption of the oral microbiota could affect the development of major systemic diseases, such as oral cancer. New research has clarified the processes by which *P. gingivalis* causes oral carcinogenesis. Muñoz-Medel M et al. show that oral infection with P. gingivalis increases the levels of lipopolysaccharides and gingipains, which induce chronic inflammation and immune evasion, thus favoring gastric carcinogenesis [98]. Wang B et al. review the complex interactions between P. gingivalis and Fusobacterium nucleatum and their impact on carcinogenesis. This study highlights the importance of understanding microbial interactions in the development of oral cancer [99].

In addition to DNA methylation, changes in histone acetylation represent another crucial way in which oral pathogenic bacteria could affect gene expression. Proteins known as histones control access to genetic material by helping to package DNA in the nucleus. By adding an acetyl group to histones, or histone acetylation, the chromatin structure can be relaxed and result in more active expression of genes related to cell proliferation [100]. Oral dysbiosis can alter histone acetylation, stimulating the expression of several oncogenic genes and increasing uncontrolled cell proliferation [101]. These changes can lead to aberrant cell division processes. Within the unbalanced oral microbiota, pathogenic bacteria can interact with histone complexes, influencing the expression of genes involved in cell proliferation and inflammation, essential factors in the development of oral cancer and other dysbiosis-related diseases [102].

## 6. Oncogenic Signaling Pathways Activated in the Context of Oral Dysbiosis

One important element linked to the activation of several intracellular signaling pathways engaged in malignant transformation is oral dysbiosis [102]. These signaling channels help to induce persistent inflammation, unchecked cell growth, and lower death rates, therefore fostering an environment fit for cancer formation. The most significant signaling channels impacted are as follows.

### 6.1. The NF-κB Pathway and Chronic Inflammation

Activated by bacteria associated with oral dysbiosis, nuclear factor kappa (NF-κB) is a major signaling pathway. Through Toll-like receptors (TLR) and Nod-like receptor (NLR) proteins, *P. gingivalis* and *F. nucleatum* activate NF-κB, thereby increasing the synthesis of IL-8 and pro-inflammatory cytokines (IL-6, IL-8, TNF-α). Through its interaction with TLR2 and TLR4, *P. gingivalis* triggers a signaling cascade within the cell that ultimately activates NF-κb [73,102,103]. The same is true of *F. nucleatum*, which activates this pathway via the NLRP3 inflammasome to produce cytokines, therefore fostering inflammation [104]. As well as abnormal cell proliferation and resistance to death, this chronic inflammatory milieu is in charge of supporting necessary mechanisms implicated in the development and dissemination of periodontal diseases and oral cancer [105]. Research has also confirmed that NF-κB controls the expression of genes connected with epithelial-mesenchymal transition (EMT), a process essential for the invasion and metastases of tumor cells [103,104,105,106]. Thus, a possible method for the prevention and treatment of oral cancer is lowering chronic inflammation utilizing NF-κB.

### 6.2. The PI3K/AKT/mTOR Pathway and Cell Survival

Involved in cell development and energy metabolism, PI3K/AKT/mTOR is another important pathway affected by oral dysbiosis. Studies on lipopolysaccharides (LPS) released by pathogenic bacteria indicate that they can activate Toll-like receptors (TLR4), therefore activating the PI3K/AKT pathway and causing too great cell proliferation and suppression of death [107,108]. PI3K/AKT/mTOR’s ongoing activation helps tumors grow and angiogenesis [109]. PI3K turns on the protein kinase AKT, which controls several biological functions, including cell survival and development. In turn, AKT phosphorylates and stimulates m TOR, a serine-threonine kinase vital for the control of cellular metabolism and protein synthesis [110]. Oral cancer is often seen with the dysregulation of this system, implying that its suppression could be a workable treatment [111].

### 6.3. The Wnt/β-Catenin Pathway and Genomic Instability

The Wnt/β-catenin signaling system is what maintains epithelial homeostasis; this route has been related to many types of cancer, including oral cancer, when it is aberrant. Recent studies reveal that *P. gingivalis* can induce the abnormal accumulative accumulation of β-catenin protein in the cytoplasm and nucleus, so controlling the expression of genes connected in proliferation (c-Myc, Cyclin D1). This deregulation favors genomic instability and more tumor invasibility [112,113]. Further important for metastases is epithelial-mesenchymal transition (EMT), which might be encouraged by dysregulation of this pathway [114].

### 6.4. The MAPK/ERK Pathway and Cell Differentiation

Control of cell proliferation and differentiation is much aided by the mitogen-activated protein kinase (MAPK/ERK) pathway. By means of its interaction with host proteins, *F. nucleatum* infection can activate MAPK signaling, hence increasing the production of pro-oncogenic transcription factors like AP-1 [115]. Thus, crucial components in cancer progression, aberrant MAPK/ERK signaling, help to explain hyperproliferation and reduced death [116,117]. Moreover, new research indicates that constant activation of this system fuels angiogenesis and chronic inflammation, therefore encouraging tumor formation [117,118].

### 6.5. MicroRNAs and Epigenetic Regulation of Signaling Pathways

Comprising between 18 and 25 nucleotides, microRNAs (miRNAs) are a type of tiny, endogenous, non-coding RNRs. Using base complementarity between the “seed” region of the miRNA and the 3′-UTR region of the target mRNA, they control gene expression concurrently by translational repression, mRNA degradation, or both processes [119]. Recent research validates the presence of a near link between miRNAs and bacteria. For instance, it has been demonstrated that gut flora directly affects host miRNAs, therefore affecting inflammatory and immunological systems [120]. Regulation of oncogenes and tumor suppressor genes depends critically on the interactions among microbiota, miRNAs, and the host [121]. Underlining the need to understand these systems for the evolution of new therapeutic techniques, disturbances of this balance can promote the onset and advancement of cancer [122].

MicroRNAs, small RNA molecules engaged in post-transcriptional control of genes, can be expressed under the influence of pathogenic oral bacteria. Recent investigations have shown that *P. gingivalis* can deregulate the expression of miRNAs, including miR-21, which inhibits pro-apoptotic genes, therefore enabling aberrant cell survival [120]. Key events in oral cancer development dysregulation of other miRNAs, including miR-155 and miR-146a, have been linked to ongoing NF-κB activation and chronic inflammation [121]. Furthermore, crucial in the control of epithelial-mesenchymal transition, a fundamental mechanism in oral cancer invasion and metastases, are miR-200 and miR-34 [122]. Apart from affecting inflammation and death, modifications in miRNAs’ expression could potentially affect therapy response. Studies on oral squamous cell carcinoma have found, for instance, a correlation between chemotherapy resistance and raised levels of miR-21 and miR-155. MiRNAs can thus be therapeutic targets in the future as well as biomarkers for diagnosis and prognosis [123,124].

## 7. Strengths and Limitations

The clinical implications of oral dysbiosis exert their effects both locally (dental caries, gingivitis, periodontitis) and systemically, contributing to cardiovascular diseases (maintenance of inflammation and malfunctions of vascular walls), diabetes mellitus (impairment of glucose metabolism and modulation of inflammation), neurodegenerative diseases (enzymes that determine amyloid formation), autoimmune diseases (production of hypercitrullinated proteins and autoantibodies), premature births (development of inflammation and premature rupture of membranes), respiratory infections (reservoir of microbes), development of chronic kidney disease (inflammatory status and release of uremic toxins) and even cancers. Thus, oral dysbiosis not only causes persistent local inflammation, but also directly affects the activation of carcinogenic molecular pathways. This complicated interaction between microorganisms and cell signaling pathways indicates a possible function of the oral microbiome in the tumorigenic process, both in its initiation and in its maintenance. Knowledge of these processes is becoming important and could pave the way for the creation of new treatment approaches, including targeting oral bacteria or the signaling pathways triggered by them.

The limitations of the study stem from the narrative nature of the review, which may introduce bias due to the inclusion of less relevant articles in the field. However, we assume that the entire study was based on both the most recent bibliographic sources and the best-rated works in the field so that we have a reference base of superior quality. In addition, we have strived to maintain objectivity by preserving the true essence of the findings from the reference studies, refraining from introducing personal interpretations or perspectives. Despite the length of the study, we believe that the careful selection and consolidation of all available information truly improves the reader’s understanding of the etiopathogenic mechanisms involved in the expression of the oral microbiota.

## 8. Conclusions

The complicated pathophysiology of oral dysbiosis consists of altered microbial balance, persistent inflammation, mucosal barrier degradation, toxin generation and host immunological dysfunction. Awareness of the relationship between dental and general health helps to build effective strategies for the prevention and treatment of oral dysbiosis. Genomic studies show that genetic elements affect the bacterial structure and the risk of oral diseases. Thus, oral dysbiosis involves genes related to glucose metabolism, immunology and detoxification. Oral microbiota interactions are controlled by the genetic diversity of the host in immunological and inflammatory reactions. Therefore, personalized oral and systemic drugs aimed at avoiding dysbiosis and preserving microbial balance should be guided by this knowledge. Thus, a comprehensive strategy that integrates environmental, epigenetic and genetic components could change the path of prevention and therapy of oral dysbiosis.

## Data Availability

Data is contained within the article.
